# 3′,5′-Di­chloro-*N*,*N*-diphenyl-[1,1′-biphen­yl]-4-amine

**DOI:** 10.1107/S2414314621010166

**Published:** 2021-10-13

**Authors:** Dinesh G. Patel, Jordan M. Cox, Branden M. Bender, Jason B. Benedict

**Affiliations:** a Department of Chemistry, the Pennsylvania State University at Hazelton, Hazelton, Pennsylvania 18202, USA; b Department of Chemistry, the State University of New York at Buffalo, Buffalo, New York 14260-3000, USA; University of Aberdeen, Scotland

**Keywords:** crystal structure, 3,5-di­chloro-1,1′-biphen­yl, tri­phenyl­amine derivative

## Abstract

The title tri­phenyl­amine derivative, C_24_H_17_Cl_2_N, featuring a 3,5-di­chloro-1,1′-biphenyl moiety has been synthesized and structurally characterized. The mol­ecular structure shows rotations of the phenyl rings in the range of 37–40° from the amine plane. In the crystal, the mol­ecules inter­act by van der Waals inter­actions.

## Abstract 2

The title compound, C_24_H_17_Cl_2_N, crystallizes with a single mol­ecule in the asymmetric unit. In the crystal, van der Waals inter­actions are responsible for the observed packing structure.

## Graphical abstract

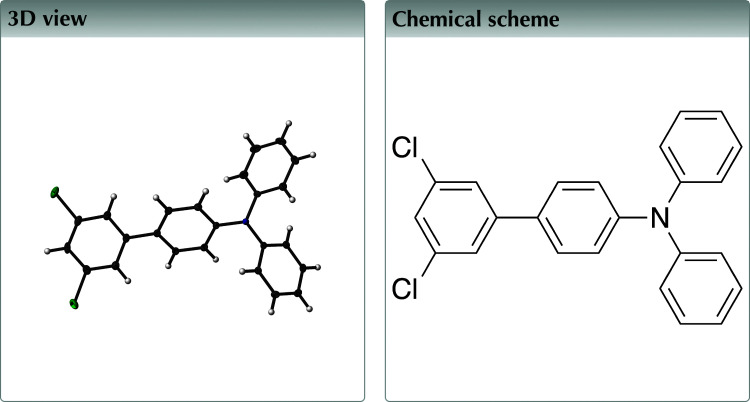

## Structure description

Owing to their electron donating ability, tri­phenyl­amine building blocks have found extensive use in organic electronic materials from polymeric (Iwan & Sek, 2011 ▸) to mol­ecular motifs (Blanchard *et al.*, 2019 ▸), including dye-sensitized solar cells (Mahmood, 2016 ▸). Mol­ecular units capable of forming *meta-*linkages, such as 1,3-dihalobenezenes, are known to organize in helical arrangements (Banno *et al.*, 2012 ▸) and have been of inter­est due to their broken conjugation (Patel *et al.*, 2011 ▸) and mechanical properties (Kandre *et al.*, 2007 ▸). Thus, the title compound, C_24_H_17_Cl_2_N, could find use as a means to impose helical design elements in organic electronic materials. Worthy of note is that the reaction proceeds well with a water-soluble palladium catalyst (Hamilton *et al.*, 2013 ▸).

**Figure 1:**
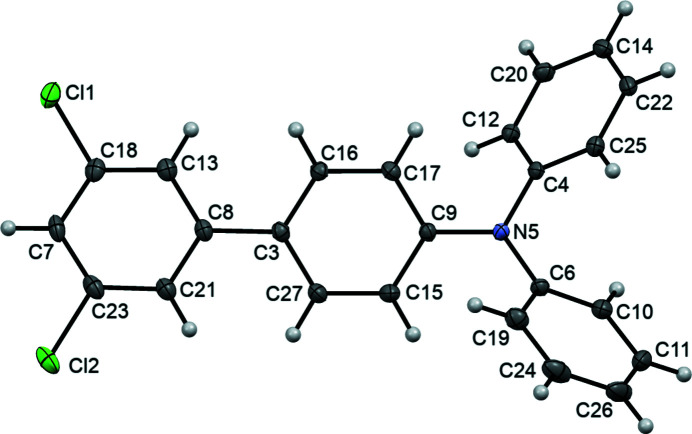
The asymmetric unit of the title compound with atom numbering. Displacement ellipsoids are drawn at the 50% probability level.

The molecular structure of the title compound (Fig. 1 ▸) shows that the tertiary nitro­gen atom adopts an almost planar environment (bond-angle sum = 358.9°). The C13–C18 and C19–C24 phenyl substituents on the amine are rotated by 38.28 (8) and 40.22 (8)°, respectively, with respect to the C1/C13/C19/N1 amine plane. The C1–C6 phenyl ring of the biphenyl moiety adjacent to the nitro­gen atom is rotated by 36.81 (8)° with respect to the same amine plane, while the C7–C12 chlorinated ring makes an angle with the amine plane of 6.04 (8)°. The dihedral angle between the C1–C6 and C7–C12 rings is 30.79 (7)°.

**Figure 2:**
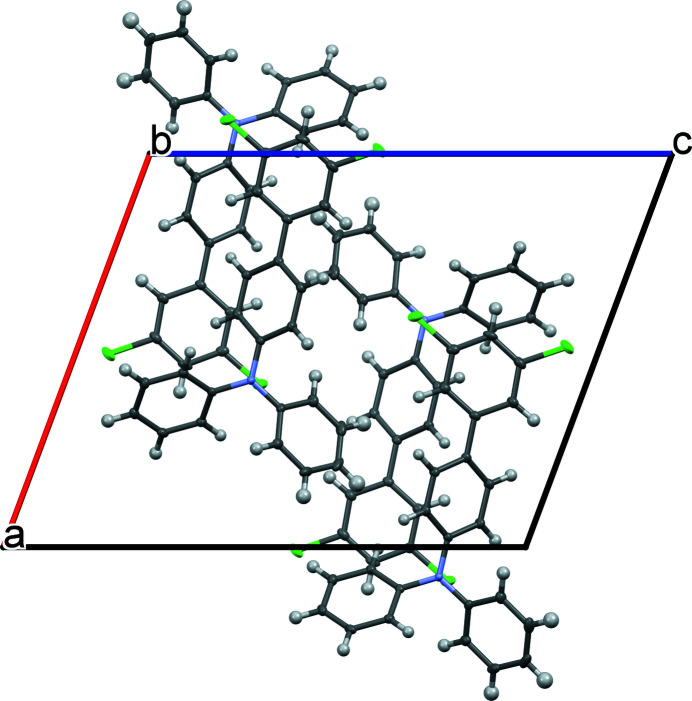
Crystal packing of the title compound viewed along [010] illustrating head-to-tail dimer formation.

**Figure 3:**
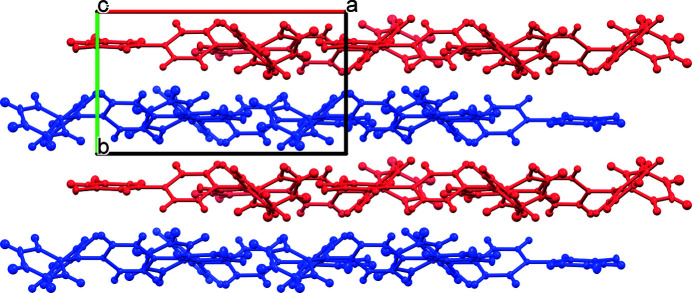
Crystal packing of the title compound viewed along [001]. Alternating layers are highlighted in blue and red.

**Figure 4:**
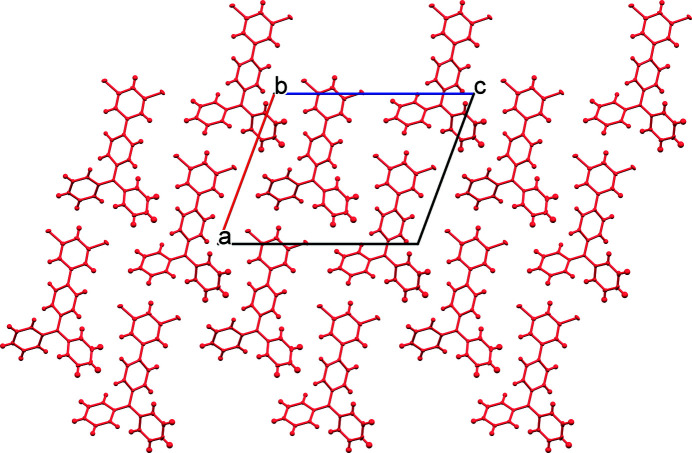
Single sheet of the title compound viewed along [010].

Mol­ecules of the title compound pack in the extended structure as head-to-tail dimers (Fig. 2 ▸). More broadly, the structure may be described as alternating sheets, which stack along [010] (Fig. 3 ▸). Defining the N5—C7 bond as the polar axis of the mol­ecule, each sheet contains a polar array of mol­ecules with their axes approximately oriented along [100] (Fig. 4 ▸). Adjacent layers exhibit similar orientations, albeit with mol­ecules pointing in the opposite polar direction. The mol­ecular packing is largely a consequence of van der Waals-type inter­actions. Although the mol­ecule contains two chlorine atoms, halogen bonding within the structure is unlikely as the shortest Cl⋯Cl contact distance of 3.74 Å is greater than the sum of the van der Waals radii for the pair (3.50 Å).

## Synthesis and crystallization

**Figure 5:**
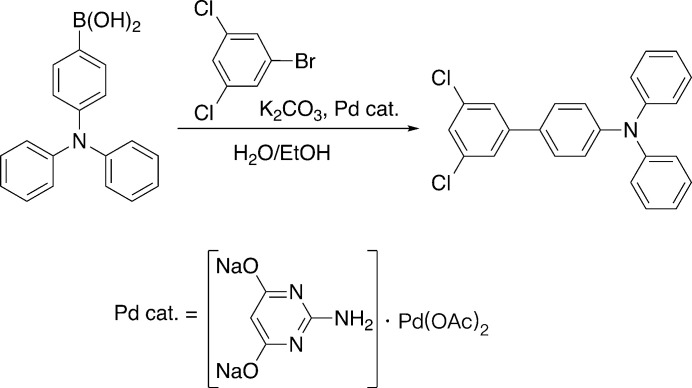
Synthetic scheme for the preparation of the title compound.

The title compound was synthesized under typical Suzuki conditions from commercially available 4-(di­phenyl­amino)­phenyl­boronic acid and 1-bromo-3,5-di­chloro­benzene as shown in Fig. 5 ▸. Briefly, the boronic acid (0.872 g, 3.02 mmol), bromide (0.681 g, 3.02 mmol), potassium carbonate (5.002 g, 36.19 mmol), water (15 ml) and ethanol (20 ml) were combined and sparged with nitro­gen for 10 minutes. The palladium catalyst (Hamilton *et al.*, 2013 ▸) (0.4 ml, 2.5 m*M* in water) was then added and the reaction heated to 80°C under nitro­gen until thin layer chromatography (silica plates, 5% ethyl acetate in hexa­ne) showed complete consumption of the starting materials. The reaction was then poured into water (50 ml) and the resulting precipitate collected by suction filtration and recrystallized from hot ethanol to afford crystals of the title compound as colorless plates (0.832 g, 71%).

## Refinement

Crystal data, data collection, and structure refinement details are summarized in Table 1 ▸.

## Supplementary Material

Crystal structure: contains datablock(s) I. DOI: 10.1107/S2414314621010166/hb4393sup1.cif

Structure factors: contains datablock(s) I. DOI: 10.1107/S2414314621010166/hb4393Isup2.hkl

Supporting information file. DOI: 10.1107/S2414314621010166/hb4393Isup3.cml

CCDC reference: 2113293

Additional supporting information:  crystallographic information; 3D view; checkCIF report

## Figures and Tables

**Table 1:** Experimental details

Crystal data	
Chemical formula	C_24_H_17_Cl_2_N
*M*_r_	390.28
Crystal system, space group	Monoclinic, *P*2_1_/*n*
Temperature (K)	90
*a*, *b*, *c* (Å)	14.5188 (11), 7.7744 (7), 18.0700 (16)
β (°)	110.4472 (18)
*V* (Å^3^)	1911.1 (3)
*Z*	4
Radiation type	Mo *K*α
μ (mm^−1^)	0.35
Crystal size (mm)	0.32 × 0.24 × 0.04

Data collection	
Diffractometer	Bruker SMART APEXII area detector
Absorption correction	Multi-scan (*SADABS*; Bruker, 2018 ▸)
*T*_min_, *T*_max_	0.677, 0.746
No. of measured, independent and observed [*I* > 2σ(*I*)] reflections	18024, 6318, 4600
*R*_int_	0.041
(sin θ/λ)_max_ (Å^−1^)	0.748

Refinement	
*R*[*F* ^2^ > 2σ(*F* ^2^)], *wR*(*F* ^2^), *S*	0.044, 0.116, 1.02
No. of reflections	6318
No. of parameters	244
H-atom treatment	H-atom parameters constrained
Δρ_max_, Δρ_min_ (e Å^−3^)	0.45, −0.30

Computer programs: *APEX3* and *SAINT* (Bruker, 2018 ▸), *olex2.solve* (Dolomanov *et al.*, 2009 ▸), *SHELXL* (Sheldrick, 2015 ▸), and *OLEX2* (Dolomanov *et al.*, 2009 ▸).

## full crystallographic data

### Crystal data

**Table:**

C_24_H_17_Cl_2_N	*F*(000) = 808
*M**_r_* = 390.28	*D*_x_ = 1.356 Mg m^−^^3^
Monoclinic, *P*2_1_/*n*	Mo *K*α radiation, λ = 0.71073 Å
*a* = 14.5188 (11) Å	Cell parameters from 6404 reflections
*b* = 7.7744 (7) Å	θ = 2.9–32.1°
*c* = 18.0700 (16) Å	µ = 0.35 mm^−^^1^
β = 110.4472 (18)°	*T* = 90 K
*V* = 1911.1 (3) Å^3^	Plate, colourless
*Z* = 4	0.32 × 0.24 × 0.04 mm

### Data collection

**Table:**

Bruker SMART APEXII area detector diffractometer	6318 independent reflections
Radiation source: microfocus sealed X-ray tube, Incoatec Iµs	4600 reflections with *I* > 2σ(*I*)
Mirror optics monochromator	*R*_int_ = 0.041
Detector resolution: 7.9 pixels mm^-1^	θ_max_ = 32.1°, θ_min_ = 1.6°
ω and φ scans	*h* = −20→18
Absorption correction: multi-scan (SADABS; Bruker, 2018)	*k* = −10→11
*T*_min_ = 0.677, *T*_max_ = 0.746	*l* = −24→27
18024 measured reflections	

### Refinement

**Table:**

Refinement on *F*^2^	0 restraints
Least-squares matrix: full	Hydrogen site location: inferred from neighbouring sites
*R*[*F*^2^ > 2σ(*F*^2^)] = 0.044	H-atom parameters constrained
*wR*(*F*^2^) = 0.116	*w* = 1/[σ^2^(*F*_o_^2^) + (0.0584*P*)^2^ + 0.262*P*] where *P* = (*F*_o_^2^ + 2*F*_c_^2^)/3
*S* = 1.02	(Δ/σ)_max_ = 0.001
6318 reflections	Δρ_max_ = 0.45 e Å^−^^3^
244 parameters	Δρ_min_ = −0.30 e Å^−^^3^

### Special details

**Table:**

Geometry. All esds (except the esd in the dihedral angle between two l.s. planes) are estimated using the full covariance matrix. The cell esds are taken into account individually in the estimation of esds in distances, angles and torsion angles; correlations between esds in cell parameters are only used when they are defined by crystal symmetry. An approximate (isotropic) treatment of cell esds is used for estimating esds involving l.s. planes.
Refinement. H atoms were placed geometrically (C—H = 0.95 Å) and refined as riding atoms with *U*_iso_(H) = 1.2*U*_eq_(C).

### Fractional atomic coordinates and isotropic or equivalent isotropic displacement parameters (Å^2^)

**Table:**

	*x*	*y*	*z*	*U*_iso_*/*U*_eq_	
Cl1	0.58639 (3)	0.75629 (5)	0.37567 (2)	0.02440 (10)	
Cl2	0.50900 (3)	0.69374 (6)	0.06326 (2)	0.03003 (11)	
C4	0.22713 (10)	0.73656 (17)	0.17719 (8)	0.0137 (3)	
C13	−0.10562 (10)	0.75089 (17)	0.21142 (7)	0.0125 (2)	
N1	−0.07602 (9)	0.73451 (16)	0.14480 (6)	0.0144 (2)	
C19	−0.14718 (10)	0.75157 (18)	0.06761 (7)	0.0141 (3)	
C10	0.53724 (11)	0.72175 (18)	0.21805 (9)	0.0190 (3)	
H10	0.605687	0.714403	0.227325	0.023*	
C7	0.33384 (11)	0.73663 (17)	0.19079 (8)	0.0147 (3)	
C1	0.02498 (10)	0.73489 (17)	0.15553 (7)	0.0130 (3)	
C20	−0.23743 (11)	0.6683 (2)	0.04815 (8)	0.0187 (3)	
H20	−0.250201	0.594839	0.085468	0.022*	
C21	−0.30879 (12)	0.6926 (2)	−0.02586 (9)	0.0253 (3)	
H21	−0.370919	0.637873	−0.038583	0.030*	
C14	−0.05678 (10)	0.65661 (18)	0.28000 (7)	0.0140 (3)	
H14	−0.005075	0.580467	0.281248	0.017*	
C8	0.40304 (11)	0.75138 (17)	0.26757 (8)	0.0160 (3)	
H8	0.381605	0.767131	0.311129	0.019*	
C16	−0.16027 (11)	0.7831 (2)	0.34467 (8)	0.0189 (3)	
H16	−0.179045	0.793924	0.389821	0.023*	
C6	0.06067 (11)	0.64239 (19)	0.10468 (8)	0.0167 (3)	
H6	0.016417	0.577499	0.062682	0.020*	
C3	0.19096 (10)	0.82689 (17)	0.22789 (8)	0.0136 (3)	
H3	0.235370	0.889506	0.270698	0.016*	
C2	0.09205 (10)	0.82738 (17)	0.21728 (8)	0.0140 (3)	
H2	0.069372	0.891289	0.252341	0.017*	
C9	0.50190 (11)	0.74299 (18)	0.27962 (9)	0.0174 (3)	
C24	−0.12794 (12)	0.8547 (2)	0.01149 (8)	0.0195 (3)	
H24	−0.066167	0.910609	0.024041	0.023*	
C15	−0.08374 (11)	0.67414 (19)	0.34618 (8)	0.0178 (3)	
H15	−0.049628	0.611252	0.392795	0.021*	
C12	0.36787 (11)	0.71894 (19)	0.12771 (9)	0.0181 (3)	
H12	0.322600	0.711890	0.075086	0.022*	
C17	−0.20905 (11)	0.87587 (19)	0.27654 (8)	0.0175 (3)	
H17	−0.261409	0.950418	0.275312	0.021*	
C11	0.46825 (12)	0.71177 (19)	0.14253 (9)	0.0197 (3)	
C23	−0.19930 (13)	0.8754 (2)	−0.06270 (9)	0.0266 (4)	
H23	−0.185790	0.944557	−0.101048	0.032*	
C18	−0.18223 (10)	0.86104 (18)	0.21019 (8)	0.0152 (3)	
H18	−0.215906	0.925670	0.164021	0.018*	
C22	−0.28971 (13)	0.7965 (2)	−0.08128 (9)	0.0295 (4)	
H22	−0.338688	0.813149	−0.131806	0.035*	
C5	0.15979 (11)	0.64454 (19)	0.11497 (8)	0.0168 (3)	
H5	0.182456	0.582695	0.079321	0.020*	

### Atomic displacement parameters (Å^2^)

**Table:**

	*U*^11^	*U*^22^	*U*^33^	*U*^12^	*U*^13^	*U*^23^
Cl1	0.0142 (2)	0.0296 (2)	0.02519 (18)	0.00058 (15)	0.00163 (14)	−0.00117 (14)
Cl2	0.0253 (2)	0.0422 (2)	0.0312 (2)	0.00160 (18)	0.02072 (17)	−0.00223 (17)
C4	0.0133 (7)	0.0140 (6)	0.0146 (5)	0.0024 (5)	0.0057 (5)	0.0020 (5)
C13	0.0108 (7)	0.0145 (6)	0.0126 (5)	−0.0027 (5)	0.0048 (5)	−0.0019 (4)
N1	0.0099 (6)	0.0230 (6)	0.0100 (4)	0.0004 (5)	0.0032 (4)	0.0001 (4)
C19	0.0133 (7)	0.0177 (6)	0.0108 (5)	0.0028 (5)	0.0037 (5)	0.0003 (5)
C10	0.0128 (8)	0.0162 (6)	0.0309 (8)	0.0017 (5)	0.0114 (6)	0.0004 (5)
C7	0.0133 (7)	0.0136 (6)	0.0191 (6)	0.0011 (5)	0.0079 (5)	0.0007 (5)
C1	0.0115 (7)	0.0158 (6)	0.0126 (5)	0.0011 (5)	0.0051 (5)	0.0018 (4)
C20	0.0158 (8)	0.0246 (7)	0.0159 (6)	0.0006 (6)	0.0059 (5)	−0.0040 (5)
C21	0.0134 (8)	0.0399 (9)	0.0197 (7)	0.0024 (7)	0.0020 (6)	−0.0111 (6)
C14	0.0120 (7)	0.0157 (6)	0.0139 (6)	−0.0009 (5)	0.0042 (5)	−0.0004 (4)
C8	0.0159 (7)	0.0142 (6)	0.0189 (6)	0.0008 (5)	0.0074 (5)	0.0014 (5)
C16	0.0169 (8)	0.0265 (7)	0.0149 (6)	−0.0041 (6)	0.0077 (5)	−0.0051 (5)
C6	0.0157 (7)	0.0207 (7)	0.0135 (6)	−0.0011 (5)	0.0049 (5)	−0.0036 (5)
C3	0.0130 (7)	0.0134 (6)	0.0147 (6)	−0.0013 (5)	0.0054 (5)	−0.0007 (4)
C2	0.0152 (7)	0.0133 (6)	0.0147 (6)	0.0013 (5)	0.0068 (5)	−0.0002 (5)
C9	0.0146 (7)	0.0138 (6)	0.0225 (6)	−0.0003 (5)	0.0049 (5)	0.0000 (5)
C24	0.0213 (8)	0.0211 (7)	0.0174 (6)	0.0026 (6)	0.0084 (6)	0.0033 (5)
C15	0.0180 (8)	0.0218 (7)	0.0129 (6)	−0.0023 (6)	0.0047 (5)	0.0003 (5)
C12	0.0169 (8)	0.0197 (7)	0.0203 (6)	0.0014 (6)	0.0098 (6)	−0.0006 (5)
C17	0.0121 (7)	0.0221 (7)	0.0195 (6)	−0.0011 (5)	0.0068 (5)	−0.0058 (5)
C11	0.0199 (8)	0.0188 (7)	0.0259 (7)	−0.0002 (6)	0.0148 (6)	−0.0007 (5)
C23	0.0336 (10)	0.0311 (8)	0.0167 (6)	0.0146 (7)	0.0110 (6)	0.0071 (6)
C18	0.0118 (7)	0.0177 (6)	0.0152 (6)	−0.0001 (5)	0.0037 (5)	−0.0005 (5)
C22	0.0251 (9)	0.0456 (10)	0.0128 (6)	0.0180 (8)	0.0005 (6)	−0.0024 (6)
C5	0.0159 (7)	0.0206 (6)	0.0153 (6)	0.0024 (5)	0.0073 (5)	−0.0017 (5)

### Geometric parameters (Å, º)

**Table:**

Cl1—C9	1.7435 (15)	C14—C15	1.3889 (18)
Cl2—C11	1.7359 (14)	C8—H8	0.9500
C4—C7	1.481 (2)	C8—C9	1.376 (2)
C4—C3	1.3945 (18)	C16—H16	0.9500
C4—C5	1.401 (2)	C16—C15	1.390 (2)
C13—N1	1.4182 (16)	C16—C17	1.389 (2)
C13—C14	1.4007 (18)	C6—H6	0.9500
C13—C18	1.3977 (19)	C6—C5	1.385 (2)
N1—C19	1.4226 (17)	C3—H3	0.9500
N1—C1	1.4105 (18)	C3—C2	1.3809 (19)
C19—C20	1.392 (2)	C2—H2	0.9500
C19—C24	1.3954 (19)	C24—H24	0.9500
C10—H10	0.9500	C24—C23	1.388 (2)
C10—C9	1.388 (2)	C15—H15	0.9500
C10—C11	1.384 (2)	C12—H12	0.9500
C7—C8	1.405 (2)	C12—C11	1.388 (2)
C7—C12	1.3985 (18)	C17—H17	0.9500
C1—C6	1.4010 (18)	C17—C18	1.3889 (18)
C1—C2	1.3968 (19)	C23—H23	0.9500
C20—H20	0.9500	C23—C22	1.380 (3)
C20—C21	1.389 (2)	C18—H18	0.9500
C21—H21	0.9500	C22—H22	0.9500
C21—C22	1.388 (3)	C5—H5	0.9500
C14—H14	0.9500		
			
C3—C4—C7	120.19 (12)	C5—C6—H6	119.6
C3—C4—C5	117.83 (13)	C4—C3—H3	119.3
C5—C4—C7	121.97 (12)	C2—C3—C4	121.48 (13)
C14—C13—N1	119.61 (12)	C2—C3—H3	119.3
C18—C13—N1	121.12 (12)	C1—C2—H2	119.7
C18—C13—C14	119.26 (12)	C3—C2—C1	120.70 (12)
C13—N1—C19	119.49 (11)	C3—C2—H2	119.7
C1—N1—C13	119.47 (11)	C10—C9—Cl1	118.44 (12)
C1—N1—C19	119.91 (11)	C8—C9—Cl1	119.14 (11)
C20—C19—N1	120.09 (12)	C8—C9—C10	122.41 (14)
C20—C19—C24	119.57 (13)	C19—C24—H24	120.1
C24—C19—N1	120.33 (13)	C23—C24—C19	119.81 (15)
C9—C10—H10	121.5	C23—C24—H24	120.1
C11—C10—H10	121.5	C14—C15—C16	120.46 (13)
C11—C10—C9	117.00 (14)	C14—C15—H15	119.8
C8—C7—C4	120.64 (12)	C16—C15—H15	119.8
C12—C7—C4	120.76 (13)	C7—C12—H12	120.2
C12—C7—C8	118.59 (13)	C11—C12—C7	119.52 (14)
C6—C1—N1	120.92 (12)	C11—C12—H12	120.2
C2—C1—N1	120.80 (12)	C16—C17—H17	119.6
C2—C1—C6	118.28 (13)	C18—C17—C16	120.81 (13)
C19—C20—H20	120.0	C18—C17—H17	119.6
C21—C20—C19	119.97 (14)	C10—C11—Cl2	118.69 (12)
C21—C20—H20	120.0	C10—C11—C12	122.51 (13)
C20—C21—H21	119.9	C12—C11—Cl2	118.79 (12)
C22—C21—C20	120.27 (16)	C24—C23—H23	119.7
C22—C21—H21	119.9	C22—C23—C24	120.60 (15)
C13—C14—H14	119.9	C22—C23—H23	119.7
C15—C14—C13	120.17 (13)	C13—C18—H18	120.0
C15—C14—H14	119.9	C17—C18—C13	119.91 (13)
C7—C8—H8	120.0	C17—C18—H18	120.0
C9—C8—C7	119.93 (13)	C21—C22—H22	120.1
C9—C8—H8	120.0	C23—C22—C21	119.74 (14)
C15—C16—H16	120.3	C23—C22—H22	120.1
C17—C16—H16	120.3	C4—C5—H5	119.5
C17—C16—C15	119.38 (12)	C6—C5—C4	120.99 (12)
C1—C6—H6	119.6	C6—C5—H5	119.5
C5—C6—C1	120.71 (13)		
			
C4—C7—C8—C9	176.90 (13)	C20—C19—C24—C23	0.9 (2)
C4—C7—C12—C11	−177.15 (13)	C20—C21—C22—C23	0.2 (2)
C4—C3—C2—C1	0.8 (2)	C14—C13—N1—C19	148.16 (13)
C13—N1—C19—C20	−45.47 (18)	C14—C13—N1—C1	−44.03 (18)
C13—N1—C19—C24	133.12 (14)	C14—C13—C18—C17	0.0 (2)
C13—N1—C1—C6	149.15 (13)	C8—C7—C12—C11	1.7 (2)
C13—N1—C1—C2	−30.74 (19)	C16—C17—C18—C13	0.3 (2)
C13—C14—C15—C16	1.0 (2)	C6—C1—C2—C3	−0.2 (2)
N1—C13—C14—C15	178.69 (12)	C3—C4—C7—C8	30.22 (19)
N1—C13—C18—C17	−179.36 (13)	C3—C4—C7—C12	−150.93 (13)
N1—C19—C20—C21	176.51 (13)	C3—C4—C5—C6	−0.5 (2)
N1—C19—C24—C23	−177.65 (13)	C2—C1—C6—C5	−0.8 (2)
N1—C1—C6—C5	179.35 (13)	C9—C10—C11—Cl2	177.49 (11)
N1—C1—C2—C3	179.69 (12)	C9—C10—C11—C12	−1.2 (2)
C19—N1—C1—C6	−43.08 (19)	C24—C19—C20—C21	−2.1 (2)
C19—N1—C1—C2	137.03 (13)	C24—C23—C22—C21	−1.3 (2)
C19—C20—C21—C22	1.5 (2)	C15—C16—C17—C18	0.0 (2)
C19—C24—C23—C22	0.8 (2)	C12—C7—C8—C9	−2.0 (2)
C7—C4—C3—C2	−179.17 (12)	C17—C16—C15—C14	−0.7 (2)
C7—C4—C5—C6	178.17 (13)	C11—C10—C9—Cl1	−179.96 (11)
C7—C8—C9—Cl1	−178.43 (10)	C11—C10—C9—C8	1.0 (2)
C7—C8—C9—C10	0.6 (2)	C18—C13—N1—C19	−32.49 (19)
C7—C12—C11—Cl2	−178.84 (11)	C18—C13—N1—C1	135.33 (14)
C7—C12—C11—C10	−0.1 (2)	C18—C13—C14—C15	−0.7 (2)
C1—N1—C19—C20	146.77 (13)	C5—C4—C7—C8	−148.45 (14)
C1—N1—C19—C24	−34.64 (19)	C5—C4—C7—C12	30.4 (2)
C1—C6—C5—C4	1.1 (2)	C5—C4—C3—C2	−0.4 (2)
